# Sexual and reproductive health service use of unmarried adolescents in Morogoro, Tanzania: insights from a cross-sectional household survey

**DOI:** 10.3389/frph.2025.1623714

**Published:** 2025-09-04

**Authors:** Baraka M. Morris, Suleiman Chombo, Connie M. Ulrich, Deodatus C. V. Kakoko

**Affiliations:** ^1^ Department of Nursing Management, Muhimbili University of Health and Allied Sciences, Dar es Salaam, Tanzania; ^2^ Department of Behavioral Sciences, Muhimbili University of Health and Allied Sciences, Dar es Salaam, Tanzania; ^3^ Department of Epidemiology and Biostatistics, Muhimbili University of Health and Allied Sciences, Dar es Salaam, Tanzania; ^4^ Biobehavioral Department, School of Nursing, University of Pennsylvania, Philadelphia, PA, United States; ^5^ NewCourtland Center for Transitions and Health, School of Nursing, University of Pennsylvania, Philadelphia, PA, United States; ^6^ Leonard Davis Institute of Health Economics, University of Pennsylvania, Philadelphia, PA, United States

**Keywords:** unmarried adolescents, adolescents' health, sexual and reproductive health, health service utilization, Tanzania

## Abstract

**Background:**

Adolescents in Tanzania represent a dynamic group that significantly influences public health trends, particularly in sexual and reproductive health. Despite the availability of adolescent-friendly sexual and reproductive health services (SRHS), their utilization remains low, especially among unmarried adolescents. This potentially exposes them to risky sexual behaviors, unwanted pregnancies, unsafe abortions, and sexually transmitted infections (STIs), including HIV. This paper examines the use of SRHS by unmarried adolescents in Tanzania and the factors associated with it.

**Methods:**

A cross-sectional household survey was conducted over eight months among 312 unmarried adolescents (15–19 years) from Morogoro Municipal and Mvomero District, Tanzania. Data collection utilized the World Health Organization (WHO)-approved Cleland questionnaire, and analysis was performed using STATA 18. To identify factors associated with service utilization, bivariate Chi-square tests and multivariable modified Poisson regression analyses were performed, with significance set at *p* < 0.05.

**Results:**

Among 312 unmarried adolescents, 18% had ever used SRH services. Use was significantly higher among those who had worked for pay (aRR = 1.98, *p* = 0.025) and those with sexual relationship experience (aRR = 2.27, *p* = 0.007).

**Conclusion:**

This paper found persistently low uptake of SRHS among unmarried adolescents in Tanzania, despite strong national policy commitments to improve access. This highlights the gap between policy and the everyday realities of adolescents, shaped by work and relationships. Effective solutions require youth-centered interventions grounded in social spaces, peer networks, and digital platforms to improve service utilization.

## Background

Adolescents represent a critical segment of the population and are at the heart of public health progress (1). Defined as individuals aged 10–19 years, they comprise 16% of the global population (2–4), with the majority concentrated in low- and middle-income countries. While Southeast Asia has the largest absolute number of adolescents, Sub-Saharan Africa (SSA) has the highest proportion, with adolescents making up nearly 25% of the region's population (3). In Tanzania, this group constitutes approximately 23% of the total population (1, 3, 5), highlighting the need for tailored health interventions. Given their substantial share, adolescents present a vital opportunity to strengthen adolescent-friendly health services, particularly in sexual and reproductive health (SRH) (3). Yet in Tanzania, this potential remains underutilized due to persistent underinvestment in adolescent health and low uptake of SRH services.

Tanzania upholds adolescents’ right to access sexual and reproductive health (SRH) services and well-being through adolescent-friendly platforms (6). Recent progress demonstrates the government's commitment to realizing this right for an often underserved age group. This commitment is critical, as adolescents’ health and well-being have historically been overlooked. Despite widespread recognition of their importance, their health needs still receive limited attention (6, 7). This neglect partly stems from an unresolved disconnect between cultural and social norms that restrict access—especially for unmarried adolescents—and adolescents’ lived realities, which strongly influence their sexual behavior (6, 8, 9). While parents are expected to guide their children, many lack the skills or confidence to discuss topics such as sex, pregnancy prevention, and risky behaviors (10–12). Conservative beliefs, religious teachings, and gender norms often discourage such conversations (11, 13, 14). These same norms stigmatize premarital sex and sexually active girls, creating significant barriers to accessing SRH services (15, 16). Consequently, unmarried adolescents frequently face judgment and discrimination when seeking contraception (17, 18), undermining their right to access the very services Tanzania seeks to uphold.

Despite national and global efforts to improve adolescent sexual and reproductive health services (SRHS), significant gaps persist in Tanzania. The country ranks 17th in Africa for adolescent fertility, yet service uptake remains low (19–21), with only about one-third of health facilities offering adolescent-friendly services (1). Access is particularly limited for unmarried adolescents, who often face stigma, moral judgment due to social norms that condemn premarital sex, and concerns about confidentiality that discourage them from seeking care (22–24). Notably, although approximately 32% of adolescents aged 15–19 are sexually active (20, 25), only 18% of those in rural areas have accessed HIV and/or SRH services (26). Modern contraceptive use among adolescent girls remains low at 19.2%. In rural areas, 86.5% use no form of family planning, and only 27% reported condom use during their last sexual encounter, increasing the risk of unintended pregnancies, unsafe abortions, and STIs, including HIV (25, 27–29). This paper examines the use of SRHS among unmarried adolescents in Tanzania and the factors associated with their uptake of services.

## Materials and methods

### Study design

A cross-sectional, quantitative household survey was employed to assess the use and determinants of SRHS utilization among unmarried adolescents aged 15–19 years in Morogoro, Tanzania. The study covered five wards representing urban (Kiwanja cha Ndege), peri-urban (Bigwa, Kihonda), and rural (Mlali, Mangae) settings within Morogoro Municipal Council and Mvomero District (see Supplementary Figure S1) (30). Morogoro Region has a population of 3,197,104, with Morogoro Municipal Council and Mvomero District accounting for 14.7% and 13.2% of this total, respectively (31). Adolescents aged 15–19 comprise 9.8% of the region's population (31). Morogoro was selected due to its persistently high teenage pregnancy rate. Although the prevalence declined from 39% in 2015/16 to 28% by 2022, it remains above the national average of 22% (20, 32). Health facilities in the region implement the nationally defined Adolescent Sexual and Reproductive Health Services (ASRHS) package, as outlined in the National Accelerated Action and Investment Agenda for Adolescent Health and Wellbeing 2021/22–2024/25 (NAIA-AHW) (7). This package includes SRH education and services aimed at preventing STIs, HIV, teenage pregnancies, and gender-based violence, while promoting adolescent nutrition, school retention, and life skills development (7).

### Sample size and sampling

The sample size was calculated using Cochran's single-proportion formula, with a 5% margin of error and a 95% confidence level (Z = 1.96). In the absence of national data on the proportion of unmarried adolescents accessing adolescent-friendly SRHS in Tanzania, the study adopted a reference value of 34.7% from a 2020 survey conducted in Rwanda among adolescents aged 10–19. This produced an initial sample size estimate of 348. A design effect of 1.5, commonly applied in multistage sampling for public health studies, was used to adjust for clustering, resulting in a sample size of 522. After accounting for a 15% non-response rate, the final target sample size was set at 600.

A rigorously structured multistage sampling framework was employed to ensure representative coverage across the Morogoro Region. The process began with the random selection of two districts: Morogoro Municipal and Mvomero. These two districts differ in setting, with Morogoro Municipal being predominantly urban and Mvomero largely ruraluding wards were then randomly selected, including three from the 29 wards in Morogoro Municipal and two from the 27 wards in Mvomero District. This was followed by the random selection of 12 streets from the urban wards and 6 villages from the rural wards. In the absence of household-level data on adolescents, eligible households were conveniently identified with the assistance of a local field guide. One adolescent was enrolled per household, with preference given to the eldest when multiple adolescents were present, to maintain consistency and minimize intra-household selection bias. Proportionate sampling was applied to determine enrollment quotas across sites, ensuring balanced representation throughout the study area. To reduce response contamination, data collection in each street or village was completed within a single day. Adolescents who were cohabiting (that is, living with a partner in a marriage-like relationship) or reported by parents or guardians to have a mental illness that could impair their ability to provide informed assent or participate meaningfully were excluded.

### Data collection

The study used the Illustrative Questionnaire for Interview-Surveys with Young People, a tool freely available for research involving adolescents, developed by John Cleland (33). Questionnaire items were selected from three sections of the original tool and renamed to address the aims of the study: Socioeconomic and family characteristics (29 items), Experience of heterosexual contact (8 items), and Experience of SRHS (15 items) The questionnaire also included items to capture ethical issues in the utilization of SRHS among adolescents. Since the original questionnaire was piloted and approved by the World Health Organization (WHO) for adolescent surveys, no further piloting was performed for the first three sections. However, because the newly added section had not been previously tested, a pilot study was conducted, resulting in a Cronbach's alpha reliability coefficient of 0.74.

Data were prospectively collected from unmarried adolescents from June 21, 2023, to February 2, 2024. First, permission to conduct the study was obtained from local authorities in the Morogoro region. A field guide was assigned to assist with visiting households in each street or village. For households with adolescents under 18, the adolescents gave their assent to participate, and their parents or legal guardians provided informed consent. For households with adolescents aged 18 or older, the adolescents provided their own informed consent without needing parental approval. However, if parents or guardians were present during data collection, they were briefly informed about the study. After obtaining consent, the adolescents were given a questionnaire designed to gather information on how they use adolescent-friendly sexual and reproductive health services in Tanzania, the factors associated with usage, and any ethical issues they encounter. Research assistants were available to help if an adolescent needed assistance and to ensure the questionnaire was fully completed. Adolescents were assured that their responses would remain confidential. Given the sensitive nature of the questions, they completed the questionnaire privately and sealed it in an envelope to maintain confidentiality. The questionnaire is available from the author on request.

The primary outcome variable was dichotomous (yes/no), indicating whether unmarried adolescents had ever accessed any SRHS. These services included sexual and reproductive health education, contraception, STI/HIV services, gynecological exams, pregnancy testing, post-abortion care, and maternal health services. Independent variables consisted of participants’ sociodemographic details: age group (15–16, 17–18, or 18–19 years), sex (male/female), residence (urban/rural), education level (primary, secondary, or college), and whether they have ever worked for pay (yes/no). Family structure variables covered living with both parents(yes/no). Sexual behavior variables included whether the participant had discussed sexual issues with their father or mother (yes/no), whether they had ever been in a sexual relationship (yes/no), and whether they had ever had sexual intercourse (yes/no).

### Data analysis

Data were analyzed using STATA version 18. Missing values were checked and addressed through imputation where necessary. Descriptive statistics (frequencies, means, and standard deviations) summarized the demographic characteristics of unmarried adolescents. Bivariate analysis using the Chi-square test assessed associations between independent variables and the use of adolescent-friendly SRH services. A modified Poisson regression model with robust standard errors was employed to identify factors influencing service utilization, selected because the outcome prevalence (17%) exceeded the 10% threshold for common events. Both univariable and multivariable models were run, with variable selection based on a *p*-value < 0.2, prior literature, confounders, and stepwise elimination, which was applied to improve model parsimony and retain only variables that contributed meaningfully to the outcome (34). The variables ‘Ever had sex’ and ‘Ever been in a sexual relationship’ showed strong multicollinearity [*r* = 0.73; *χ*²(1, *N* = 312) = 166.92, *p* < 0.001], prompting exclusion of the former*.* Results were reported as crude (RR) and adjusted risk ratios (aRR), with significance set at *P*-value < 0.05. The model's goodness of fit was assessed using Pearson's chi-square test (*χ*² = 23.98, df = 7, *p*-value = 0.001) and binned residual plots, confirming residuals were randomly distributed within ±2 SD, thereby supporting a good fit.

### Ethical consideration

Ethical clearance was obtained from the MUHAS Institutional Review Board (MUHAS-IRB) with IRB No. MUHAS-REC-03-2021-521 and from the National Health Research Ethics Committee (NaTHREC) under reference No. NIMR/HQ/R.8c/Vol.1/2371. Subsequently, permission to conduct the study was granted by the Local Government Authorities in the Morogoro region, beginning with the regional administrative secretary's office, followed by the Morogoro and Mvomero district administrative secretary's offices, the Morogoro Municipal and Mvomero District Executive Directors’ offices, ward offices, and finally the village or street offices. Informed consent was provided and signed accordingly. For adolescents under 18 years of age, assent was obtained after providing adequate information about the research tailored to their level of maturity, and then a parent or legally authorized representative was asked to provide informed consent. For adolescents aged 18 or 19 years, informed consent was requested directly from them while observing the community's cultural views on consent. The confidentiality of participants’ data was strictly upheld; confidential identification numbers replaced the names of unmarried adolescents. Each adolescent was also given an envelope to seal their completed tools, ensuring confidentiality. Data are securely stored on a password-protected computer accessible only to the research team. Completed questionnaires and consent/assent forms are kept in separate locked cabinets to ensure confidentiality and data protection.

## Results

### Sociodemographic profile of unmarried adolescent respondents

Out of 604 adolescents recruited, 312 unmarried adolescents completed and returned the questionnaire, yielding a response rate of 51.7%. Participants had a mean age of 17 years (SD = 1.4), with 58% under the age of 18 (Table 1). The majority were female (58%), resided in urban areas (75%), and had attained secondary education or higher (69%). In addition, 83% had never worked for pay, and half lived with both parents. Fathers were far less likely than mothers to engage in sexual health discussions with their adolescents (21% vs. 51%). Regarding sexual behavior, approximately 35% had ever been in a romantic relationship, about a quarter had ever had sexual intercourse, and only 18% had ever used SRHS.

**Table 1:** Sociodemographic and sexual behavior characteristics of 312 unmarried adolescents and their utilization of SRHS in Morogoro, Tanzania (2023).

Variables	Category	*N* (%)	SRHS No (%)	SRHS Yes (%)	*P*-value
		312 (100)	256 (82.1)	56 (17.9)	
Age groups (Years)	Under 18	182 (58.3)	154 (84.6)	28 (15.4)	0.163
	18–19	130 (41.7)	102 (78.5)	28 (21.5)	
Sex	Female	180 (57.7)	146 (81.1)	34 (18.9)	0.613
	Male	132 (42.3)	110 (83.3)	22 (16.7)	
Residence	Urban	233 (74.7)	193 (82.8)	40 (17.2)	0.537
	Rural	79 (25.3)	63 (79.7)	16 (20.3)	
Education level	Primary	97 (31.1)	79 (81.4)	18 (18.6)	0.851
	Secondary/College	215 (68.9)	177 (82.3)	38 (17.7)	
Ever worked for pay	No	260 (83.3)	222 (85.4)	38 (14.6)	**0**.**001**
	Yes	52 (16.7)	34 (65.4)	18 (34.6)	
Live with both parents	No	143 (45.8)	117 (81.8)	26 (18.2)	0.921
	Yes	169 (54.2)	139 (82.3)	30 (17.7)	
Ever discussed sex with father	No	246 (78.8)	201 (81.7)	45 (18.3)	0.760
	Yes	66 (21.2)	55 (83.3)	11 (16.7)	
Ever discussed sex with mother	No	152 (48.7)	125 (82.2)	27 (17.8)	0.934
	Yes	160 (51.3)	131 (81.9)	29 (18.1)	
Ever been in sexual relationship	No	203 (65.1)	179 (88.2)	24 (11.8)	**0**.**000**
	Yes	109 (34.9)	77 (70.6)	32 (29.4)	
Ever had sex	No	231 (74.0)	204 (88.3)	27 (11.7)	**0**.**000**
	Yes	81 (26.0)	52 (64.2)	29 (35.8)	

Bold values indicate *p*-values from Chi-square tests that are statistically significant.

Significant associations were observed among adolescents who had ever worked for pay (*χ*² = 11.77, *p* = 0.001), been in a sexual relationship (*χ*² = 14.81, *p* < 0.001), and had sexual intercourse (*χ*² = 23.68, *p* < 0.001) (Table 1). No significant associations were found with age, sex, education level, religious affiliation, family structure, or parental communication about sex.

### Factors associated with SRHS use among unmarried adolescents

The final modified Poisson regression analysis of two key behavioral variables, namely ‘ever worked for pay’ and ‘ever been in a sexual relationship’ were included to assess their independent associations with the use of SRHS, after adjusting for key covariates such as age, sex, residence, and education level (Table 2). Adolescents who had ‘ever worked for pay’ were nearly twice as likely to utilize SRHS compared to those who had not (aRR = 1.98, *p* = 0.025). Similarly, those who had ever been in a sexual relationship were more than twice as likely to use SRHS (aRR = 2.27, *p* = 0.007).

**Table 2:** Modified poisson regression analysis of factors associated with SRHS use among 312 unmarried adolescents in Morogoro, Tanzania (2023).

Variable	RR	95% CI	*P*-value	aRR	95% CI	*P*-value
Age (Years)	1.15	0.94–1.40	0.179	0.99	0.79–1.24	0.962
Sex						
Fermale (Ref.)	1.00	–	0.647	1.00	–	0.378
Male	0.88	0.52–1.51		0.78	0.45–1.35	
Residence						
Urban (Ref.)	1.00	–	0.576	1.00	–	0.971
Rural	1.18	0.66–2.11		1.01	0.55–1.85	
Education level						
Primary (Ref.)	1.00	–	0.865	1.00	–	0.728
Secondary/College	0.95	0.54–1.67		0.90	0.49–1.65	
Ever worked for pay						
No (Ref.)	1.00	–	**0**.**003**	1.00	–	**0**.**025**
Yes	2.37	1.35–4.15		1.98	1.09–3.60	
Ever been in sexual relationships						
No (Ref.)	1.00	–	**0**.**001**	1.00	–	**0**.**007**
Yes	2.48	1.46–4.22		2.27	1.25–4.13	

Bold values indicate *p*-values from both univariable and multivariable analyses that are statistically significant.

## Discussion

This paper found low utilization of SRHS among unmarried adolescents in Tanzania, despite strong national and regional commitments to improve access. Only 18% of respondents reported using SRHS, well below the One Plan III (2021–2026) target of increasing uptake from 30% to 80% (35, 36). Higher utilization was observed among adolescents who had worked for pay or been in intimate relationships, highlighting the influence of socioeconomic and experiential factors. Existing studies attribute low uptake to barriers such as inadequate provider training, stigma, restrictive social norms, and concerns about confidentiality (37, 38). In contrast, countries such as Ethiopia have achieved greater uptake through youth-friendly services, school-based programs, peer education, non-clinical outreach, and strong political support (39–42). To achieve similar progress, Tanzania must assess if current policy efforts match adolescents’ actual experiences and focus on investing in community-based, adolescent-centered programs (43–45).

Adolescent employment is a double-edged factor in SRHS utilization, enhancing access while heightening vulnerability. Adolescents who have ever worked for pay are nearly twice as likely to use SRHS compared to those who have not, likely due to increased financial capacity to afford service-related costs. However, studies also associate adolescent employment with higher rates of sexual activity, particularly among those in informal sectors (46, 47). In Tanzania, out-of-school adolescents commonly engage in informal work such as domestic labor, street vending, transporting goods, assisting at construction sites and in food service, and operating commercial motorcycle transport (boda boda). These roles often foster premature autonomy, reduce parental oversight, and increase exposure to adult behaviors and related sexual health risks (46). To optimize SRHS uptake and reduce these risks, programs should adopt tailored non-clinical approaches, particularly community-based peer education and mobile outreach that reflect the mobility and lived experiences of working adolescents (42).

Sexual or intimate relationship experience is a key driver of SRHS utilization among unmarried adolescents, reflecting the influence of relational factors on health-seeking behavior. Adolescents with such experiences are more than twice as likely to seek care compared to their peers without similar exposure. This pattern is well-documented across sub-Saharan Africa, with adolescents often turning to SRHS reactively due to concerns about pregnancy or STI/HIV exposure (48–51). For instance, 40.5% in Ethiopia and 54.1% in Nigeria of sexually active unmarried adolescents reported having ever used emergency contraception (52, 53). Such post-exposure engagement highlights a missed opportunity for preventive care and early education. The heightened vulnerability of sexually active adolescents to adverse reproductive health outcomes underscores the need to shift from a reactive model to a proactive, developmentally appropriate approach. Rather than waiting for sexual or intimate relationship experience to drive care-seeking, interventions should anticipate risk and engage adolescents earlier, through strategies attuned to their evolving social contexts, emotional needs, and developmental stages (28, 43, 54).

Despite widespread evidence linking sociodemographic factors to SRHS uptake, this study found no significant association between age, sex, residence, or education and adolescents’ use of these services. Previous research consistently associates older age, female sex, and higher education with increased service use (43, 54). Recent studies and the Tanzania Demographic and Health Survey (TDHS) data show that sex and age are linked to sexual activity, which exposes adolescents earlier to sexual risks and increases their need for services (28, 32). The absence of expected associations in this study may result from contextual factors such as sample composition or measurement differences. Additionally, unmeasured influences such as cultural norms, service availability, and peer dynamics likely have a stronger role in shaping adolescents’ health-seeking behavior, highlighting areas for further research and targeted intervention.

### Strengths and limitations

This household-based survey captured a diverse group of unmarried adolescents, both in- and out-of-school, enhancing its real-world relevance. However, the use of a standardized WHO-approved tool without local piloting, combined with a self-administered paper survey format, may have contributed to the modest response rate, albeit acceptable. Future studies should consider different methodological approaches that might be better suited for adolescents (online). In the absence of national benchmarks for SRHS use among Tanzanian adolescents, proxy data from a Rwandan survey of adolescents aged 10–19 were used, while this study focused on those aged 15–19, introducing a minor age-related contextual gap. Lastly, the cross-sectional design limits causal inference but provides a critical snapshot of service use patterns and influencing factors. Future research should focus on longitudinal designs to better understand unmarried Tanzanian adolescents’ motivations for using or avoiding SRHS and factors supporting continued use.

## Conclusion and recommendations

This paper found persistent low uptake of SRHS among unmarried adolescents in Tanzania, despite strong national policy commitments to improve access. This underscores a critical gap between policy intent and the everyday realities that shape adolescent behavior. The findings shift attention from conventional demographic indicators to adolescents’ everyday experiences, revealing how work and relational exposure more directly shape SRHS-seeking behavior. Effective adolescent SRHS programs should be proactive, accessible, and grounded in adolescents’ lived social spaces. Recommended approaches include:

• Delivering services in adolescents’ familiar settings such as schools, sports venues, and youth clubs;
• Leveraging digital platforms and peer networks to enhance reach and relevance;
• Implementing mobile outreach clinics targeting working adolescents;
• Integrating school-based SRH education supported by digital apps; and
• Promoting peer-led community engagement initiatives that foster trust and sustained participation.

Further research into contextual influences, including mobility patterns and peer dynamics, can inform the design of more responsive and impactful interventions.

## Data Availability

The raw data supporting the conclusions of this article will be made available by the authors, without undue reservation.
