# Supplementary figures and images for: Sexual and reproductive health service use of unmarried adolescents in Morogoro, Tanzania: insights from a cross-sectional household survey
Source: Front Reprod Health. 2025 Sep 4;7:1623714. doi: 10.3389/frph.2025.1623714 (PMC12447904; doi:10.3389/frph.2025.1623714)

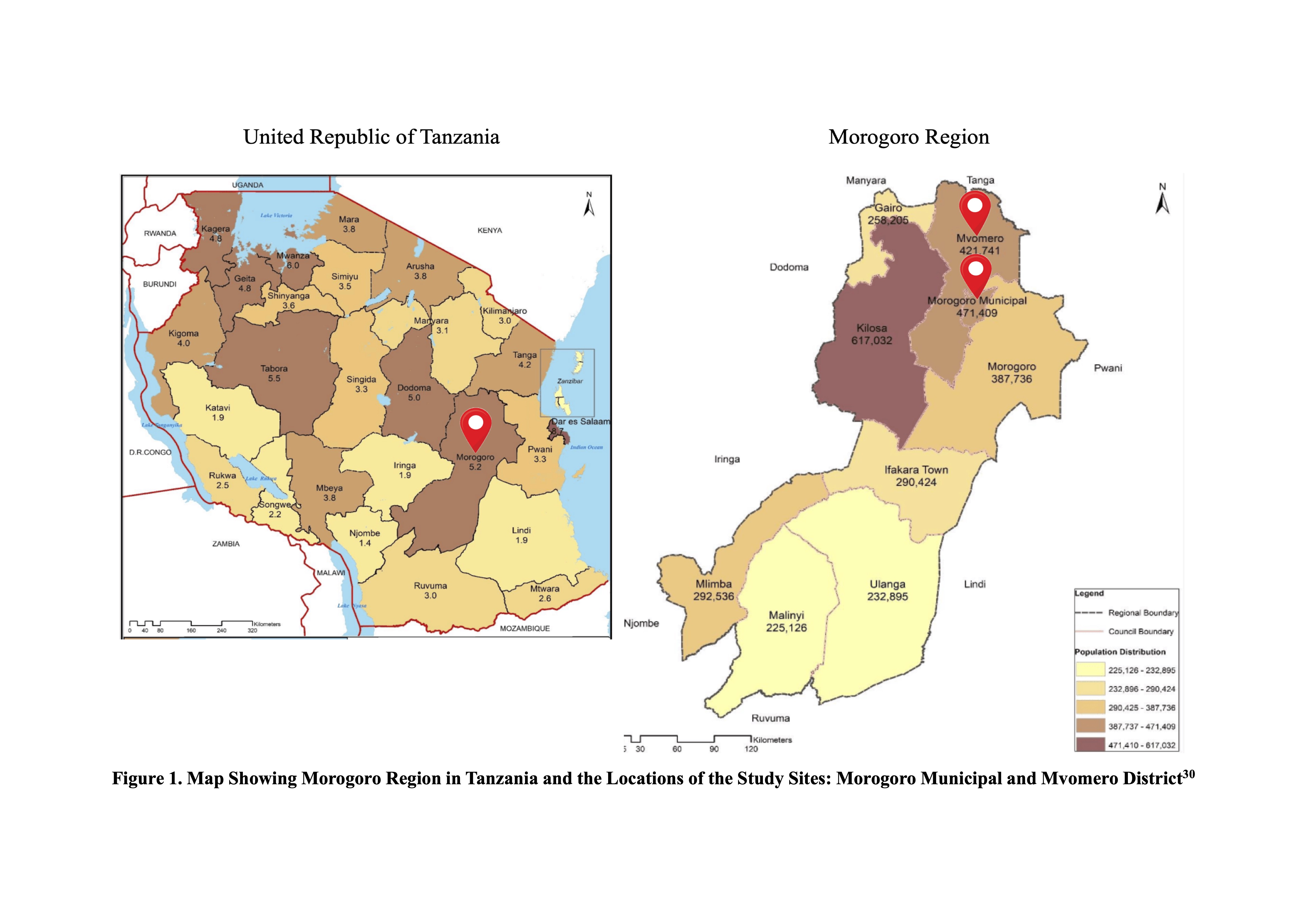

Supplement: Supplementary file 1 [file Image1.jpeg]
